# Comparative Merits of Vaccination—Old and New

**Published:** 1902-04

**Authors:** John Hauenstein

**Affiliations:** Buffalo, N.Y.


### Comparative Merits of Vaccination—Old and New.

By JOHN HAUENSTEIN, M.D., Buffalo, N.Y.

THE literature of smallpox and vaccination can be counted by the wagon load, and yet I cannot forego the temptation of adding one mite more to its bulk.

About a generation ago or less, a phantom made its appearance in the realm of smallpox and vaccination, which seemed so portentous as to terrify many physicians, for they viewed the monster as the coming syphiliser of mankind. Thereupon a hue and cry was heard against vaccination with humanised virus, although it had been considered one of the greatest blessings to man for nearly two-thirds of a century, as a safe and reliable preventive of smallpox. The result was the introduction of the vaccination with bovine virus, and since it was sometimes difficult to procure fresh humanised virus and a little troublesome, whereas bovine virus was as easily procured as 10 cents worth of candy from a candy store, it was readily adopted.

Vaccination with bovine virus having been in use a number of years, in fact so long already that the great majority of physicians now practising in this city have no recollection of the vaccination with humanised virus when used exclusively in this locality, and consequently have had no opportunity to witness a comparison of the merits of the two.

But the tempest which for a time caused such dire and portentous apprehension, based largely upon an imaginary conception, begins now to calm and a reaction commences to be perceptible, although as yet confined to the thoughtful and noiseless, but already numerous workers in the profession.

I quote from an exhaustive article written by Samuel W. Abbott, although a pro-bovine virus partisan, in the Reference Handbook of the Medical Sciences, which serves me as text in part of what I will say:

Reasons for preferring bovine virus stated by physicians, in 1881-1882: Freedom from transmitting other diseases; because of popular prejudices; greater protective power; greater uniformity of result; greater certainty; convenience.

Reasons stated for preferring humanised virus: Greater uniformity of results; less severe local and constitutional effects, with equal or greater, or proved protective power; greater promptness of action; greater reliability; freedom from serious complication.

I will in the following remarks endeavor to show up some of the weakness of the laudatory reasons for bovine virus vaccination.

The French Academy, in 1830, proclaimed:

As regards the communication of infectious or contagious diseases: Vaccinate without fear, for the vaccine virus taken from subjects afflicted with complaints susceptible of communication by contagion, such as syphilis, does not convey such germs in any case.

However, this proclamation, some years later, when the opposition mentioned appeared and terrified them, was reconsidered, found to be a little too bold, and changed.

I quote again from Abbott's article in the Reference Handbook:

Dr. Seaton, after carefully revising the different cases on record said of them: none of the alleged cases, then, have established in my opinion, that syphilis has ever been imparted in due and proper performance of vaccination with unmixed lymph of a genuine vaccine vesicle.

Again, "Dr. Foster, in his report to the American Public Health Association, also gives a list of experimenters who have purposely vaccinated with lymph from syphilitic vaccinifers in order to test the question of possibility of transmission, in over 300 vaccinations in every case the attempt to transmit syphilis was unsuccessful.

The aforementioned observations and experiments lead me to infer that the realm of the bugbear of syphilis and other infectious diseases which were assumed to be communicable by vaccination with humanised virus, has been successfully invaded and overcome.

The advocates of vaccination with humanised virus are now prepared to relate a few comprehensive truths, and arraign and prefer charges against the pro bovine virus partisans, based upon demonstrable facts and not upon hypothesis.

Quite a number of physicians in this city who had ample opportunities to witness the immediate results of bovine virus vaccination, have informed me of their observations and conclusions. They tell me that in schools where a number of children are sometimes vaccinated, who are to present themselves subsequently at a certain time for inspection of the vaccine results, a very large number showed a blurred and indistinguishable eruption, having a resemblance in some cases to what used to be called crusta lactea, observed at times on the heads and faces of infants, a variety of eczema; or an eruption such as would puzzle a dermatologist to classify. Other physicians have reported cases of destruction of the tissues to a considerable depth by sphacelus or gangrene. It has also been observed in many cases that phlegmonous erysipelas, swelling of the whole arm resulted. It is also known that children have died in this city from the effects of vaccination, and an English physician goes so far as to publish a statement that every week there is a death reported from vaccination in England. This statement, however, might be accepted with some allowance.

That cases may and doubtless have occurred of bad results and even deaths when vaccination with humanised virus was exclusively in use can be admitted, the most inconsiderable laceration of the skin may in rare cases result in poisoning of the blood, it is the frequency of such occurrences that turns the scale.

In consideration of the above mentioned facts of bovine virus vaccination, is it strange that some physicians are reluctant to vaccinate with the virus now in use, and if they can honorably escape the responsibility, will turn the subject over to some one else? Moreover, is it not plain why so many persons should be averse or apprehensive and even dread to be vaccinated when required?

I have vaccinated many thousands with humanised virus during my life with results far more satisfactory as compared with the above observations. I vaccinated almost invariably from the inspissated lymph or crust, for I could never comprehend the sophistry of preference of vaccination from arm to arm. Then we had no fears of germs communicating infectious diseases for bacteriology was yet an unknown stranger to us. Such apprehensions were never entertained by any one when vaccination with humanised virus was the exclusive practice, and how simple and harmless it was, one might infer from the fact that I was permitted to vaccinate when yet a boy, about 63 years ago. I vaccinated my first born child within twenty-four hours of its birth for fear of infection from me, having quite a number of small-pox patients on hand. To-day some anxiety would be felt as to its effect on so young a subject.

The almost invariable result was a pustule with an areola around it, beautifully picturesque, if I may be permitted the expression; only in very rare cases, and these in subjects of a marked strumous habit, would a blurred or indistinguishable eruption result.

In 1848 we had quite an epidemic of smallpox in this city, considering its limited population at the time, and I had a number of the smallpox patients to visit daily. The health authority at that time was rather lax in the enforcement of sanitary rules and consisted in almost all cases in a card with the name "smallpox" nailed on the house occupied by the patient; only exceptionally the afflicted, who had no relatives or friends to take charge of him, was sent to the pesthouse.

When called to a smallpox patient the first thing I did was to assemble all the occupants of the house in which I found the patient and then vaccinate them all with humanised virus, for we had no other at that time, and as soon as I could detect the least redness on the vaccinated spot, I was so confident that I could guarantee the subject against an attack of smallpox.

It is hardly necessary to expose the weakness and futility of "the reason for preferring bovine virus" which relates to "greater uniformity of result, greater certainty." My experience of vaccination with humanised virus in the above mentioned epidemic of smallpox, convinced me of its absolute "uniformity of result and certainty," and my reliance and confidence remain unshaken to this day. I am equally convinced that bovine virus has no claim to preference, and I doubt whether it is the equal of humanised virus.

That virus taken from a calf or heifer will not communicate syphilis, is self evident, that it has been and can be conveyed with humanised virus, has not been proved and demonstrated satisfactorily, since the evidence of the charge is largely circumstantial and hearsay, and the witness the laity, and if a case could be incontrovertibly substantiated, it would doubtless show upon investigation gross carelessness and negligence on the part of the vaccinator.

Duty requires a vaccinator to be conscientious in taking lymph from a vaccinifer, and lymph taken from a healthy child of healthy parents, the impossibility of the conveyance with it of syphilitic or any other infection must be apparent.

The claim of "greater protection power" of bovine virus vaccination in the face of past experience, is simply absurd, for at no time have reports of epidemics of smallpox in so many parts of the world been more damaging to such claim as now. It required doubtless many years after Jenner introduced vaccination before it was generally adopted; however, in whatever country or locality established, there is no evidence at command that epidemics of smallpox prevailed oftener then than now with bovine virus vaccination and I much doubt if as often, notwithstanding that our vaccination was, formerly, considered a protection for life and revaccination was not considered necessary until in comparatively recent times.

There is no evidence that the technic of vaccination has been changed in recent times, to which part of the bad results could be attributed, yet since vaccinations are not always, nor have they always been, performed with much precaution, it might be considered a service to state the observation of a physician of large practice, who tells me that since he lacerated the skin not deeper than to the corium, or perhaps more generally comprehensible, very superficially, he had better and more satisfactory results.

It remains to be said that genuine vaccination is an absolute protection against smallpox, whatever may be said by antivaccinist or medical cranks with their sophistries to the contrary, for it has saved millions of lives since its introduction. And if general vaccination could be strictly enforced upon the entry and first appearance of a case of smallpox, no isolation, no pesthouses would become necessary and thousands could be saved to a community.

Wernher zur Impffrage (on the question of vaccination) says:

Before vaccination smallpox had become a perennial disease which never entirely ceased in one year, and every 3 to 5 years became a great epidemic. In non-epidemic years 1-10 of all mortality was caused by smallpox; in epidemic years one-half.

I would hail with delight the reintroduction and adoption of the old method of vaccination with humanised virus, and that it can again be introduced with virus long ago in use is unquestionable, for there are many localities yet in Europe, where humanised virus is used, exclusively, and from which places it might be imported, if necessary.

309 Elmwood Ave.

